# Supplementary figures and images for: CD56-mediated activation of human natural killer cells is triggered by Aspergillus fumigatus galactosaminogalactan
Source: PLoS Pathog. 2024 Jun 18;20(6):e1012315. doi: 10.1371/journal.ppat.1012315 (PMC11216564; doi:10.1371/journal.ppat.1012315)

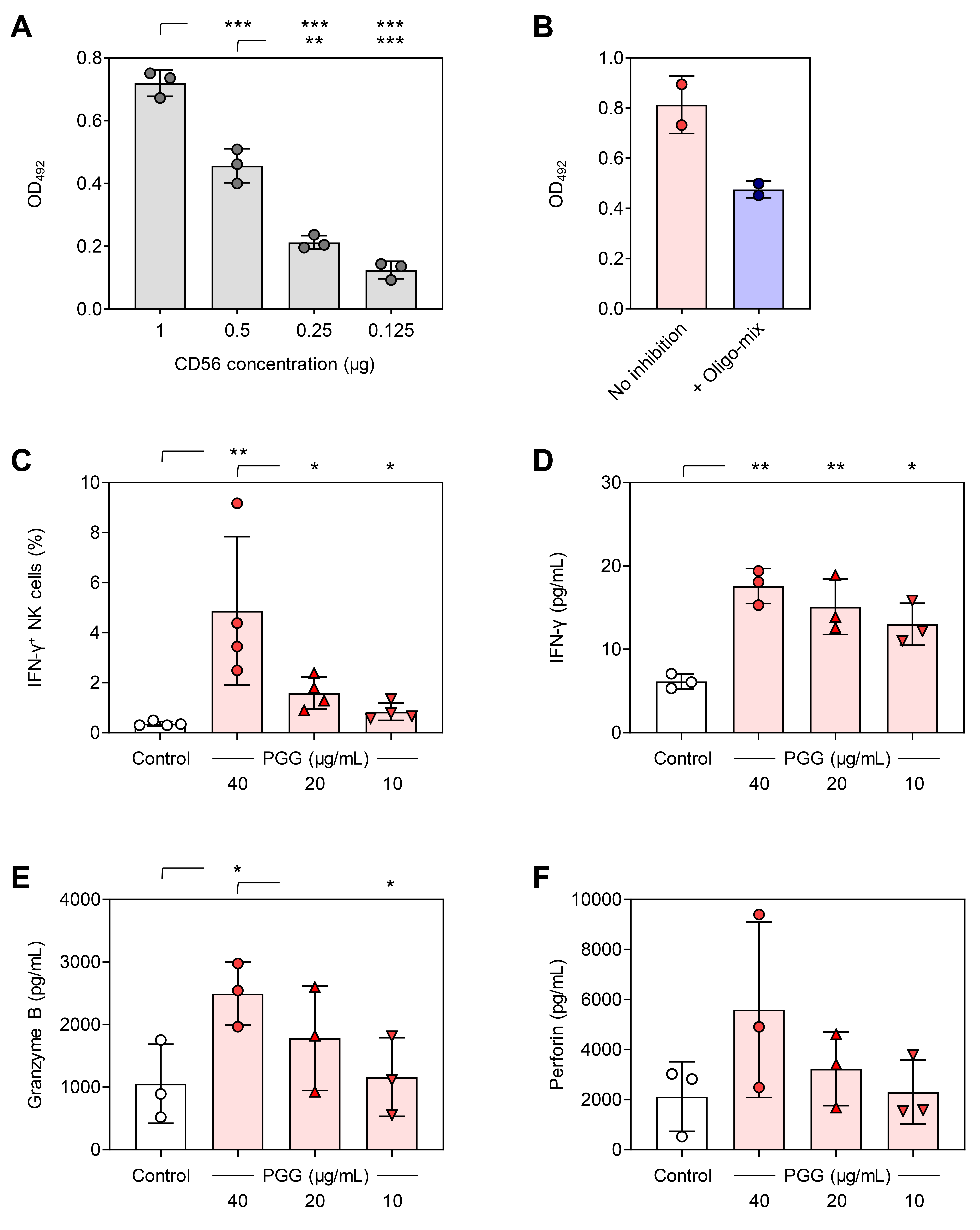

Supplement: S1 Fig — (A) Concentration dependent interaction of CD56 with galactosaminogalactan (GAG) N = 3 technical replicates, with two batches of GAG. (B) CD56 binding to GAG, with and without pre-incubation with GAG oligosaccharide fractions. N = 2 technical replicates. (C) Intracellular IFN-γ levels of naïve NK cells (Control) and NK cells stimulated for 24 h with different concentrations of urea-insoluble galactosaminogalactan (PGG). Representative histograms and data for 4 independent donors are shown. Release of IFN-γ (D), granzyme B (E) and perforin (F) by naïve NK cells (Control) and NK cells stimulated for 24 h with different concentrations of PGG. N = 3 independent donors. Columns and error bars indicate means and standard deviations, respectively. Repeated measures one-way ANOVA with Tukey’s post-hoc test. * p < 0.05, ** p < 0.01, *** p < 0.001. (TIF) [file ppat.1012315.s001.tif]

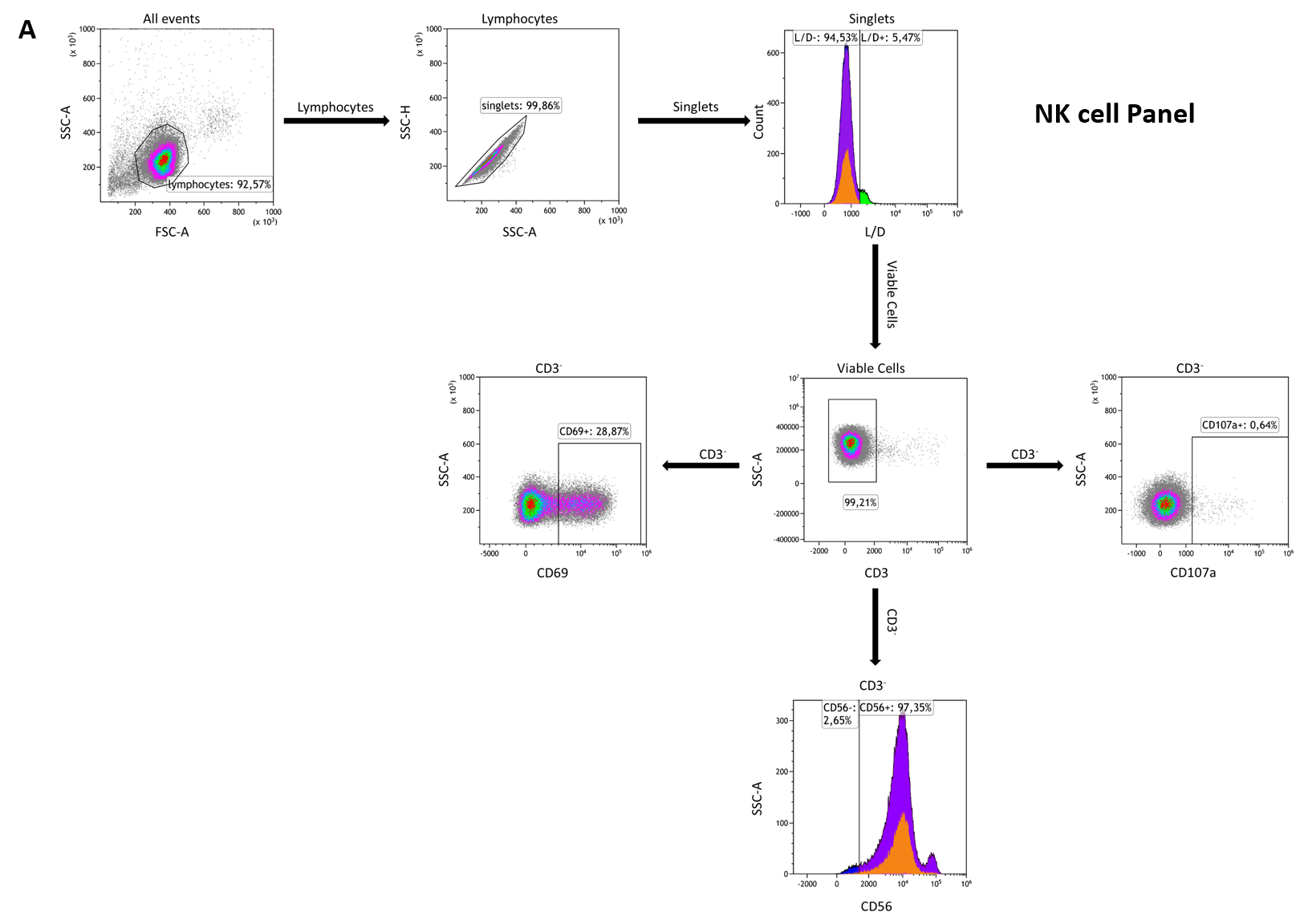

Supplement: S2 Fig — Isolated Lymphocytes are selected according to their forward and side scattering and single lymphocytes were gated based on SSC-A, and SSC-H properties. Dead lymphocytes were excluded by Live/Dead staining. Natural killer (NK) cells were identified as CD3-. Positive cells for NK cell surface marker CD56, for activation markers CD69, and degranulation marker CD107a were gated within each CD3- NK cell population. Fluorescence positivity was determined for all markers. (TIF) [file ppat.1012315.s002.tif]

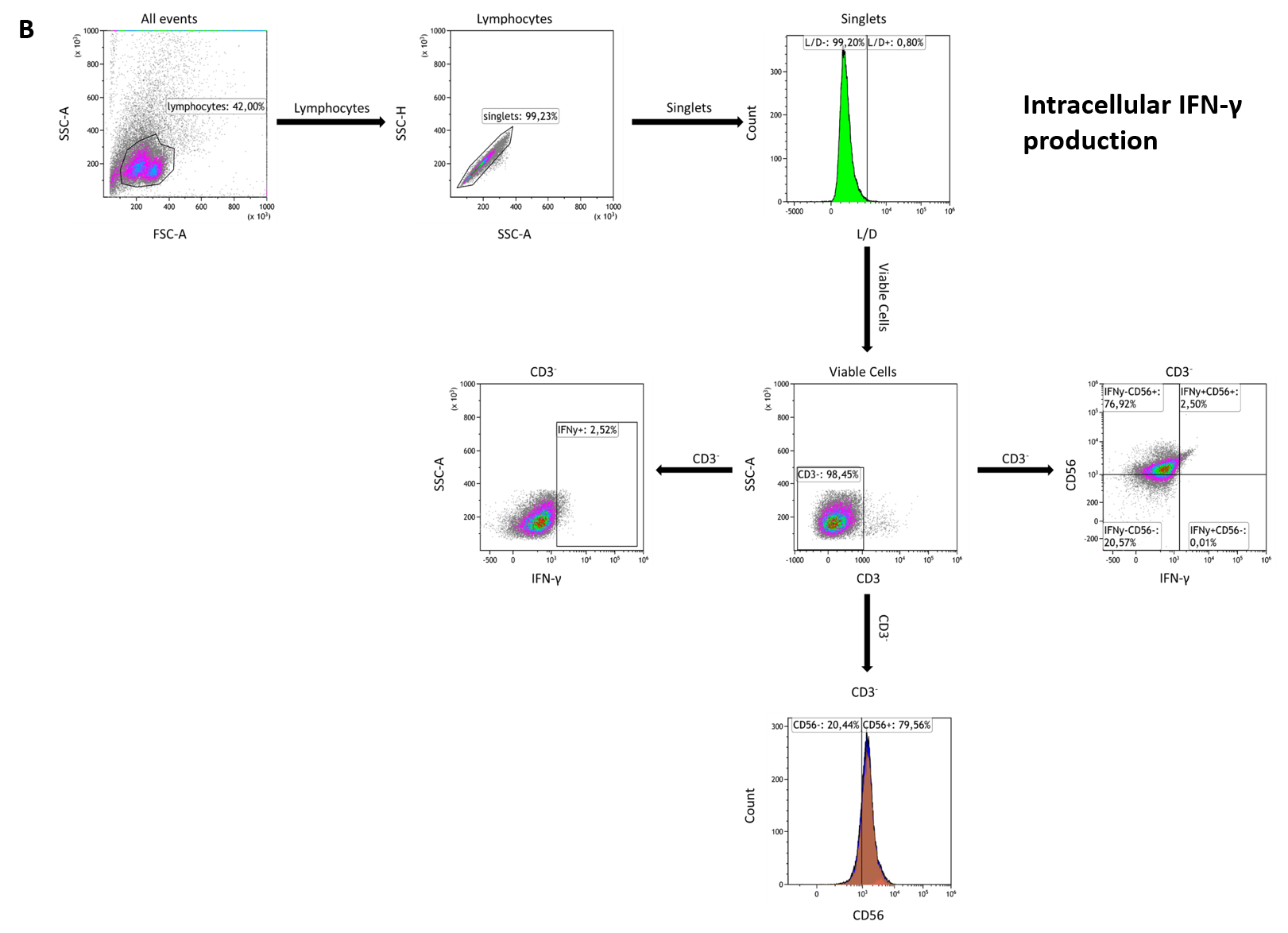

Supplement: S3 Fig — Lymphocytes are selected according to their forward and side scattering and single lymphocytes were gated based on SSC-A, and SSC-H properties. Dead lymphocytes were removed from the living cell population by Live/Dead staining. Natural killer (NK) cells were determined as CD3-. CD3- are further characterized regarding expression of the NK cell surface marker CD56, and intracellular IFN-γ levels. Fluorescence positivity was determined for all markers. (TIF) [file ppat.1012315.s003.tif]

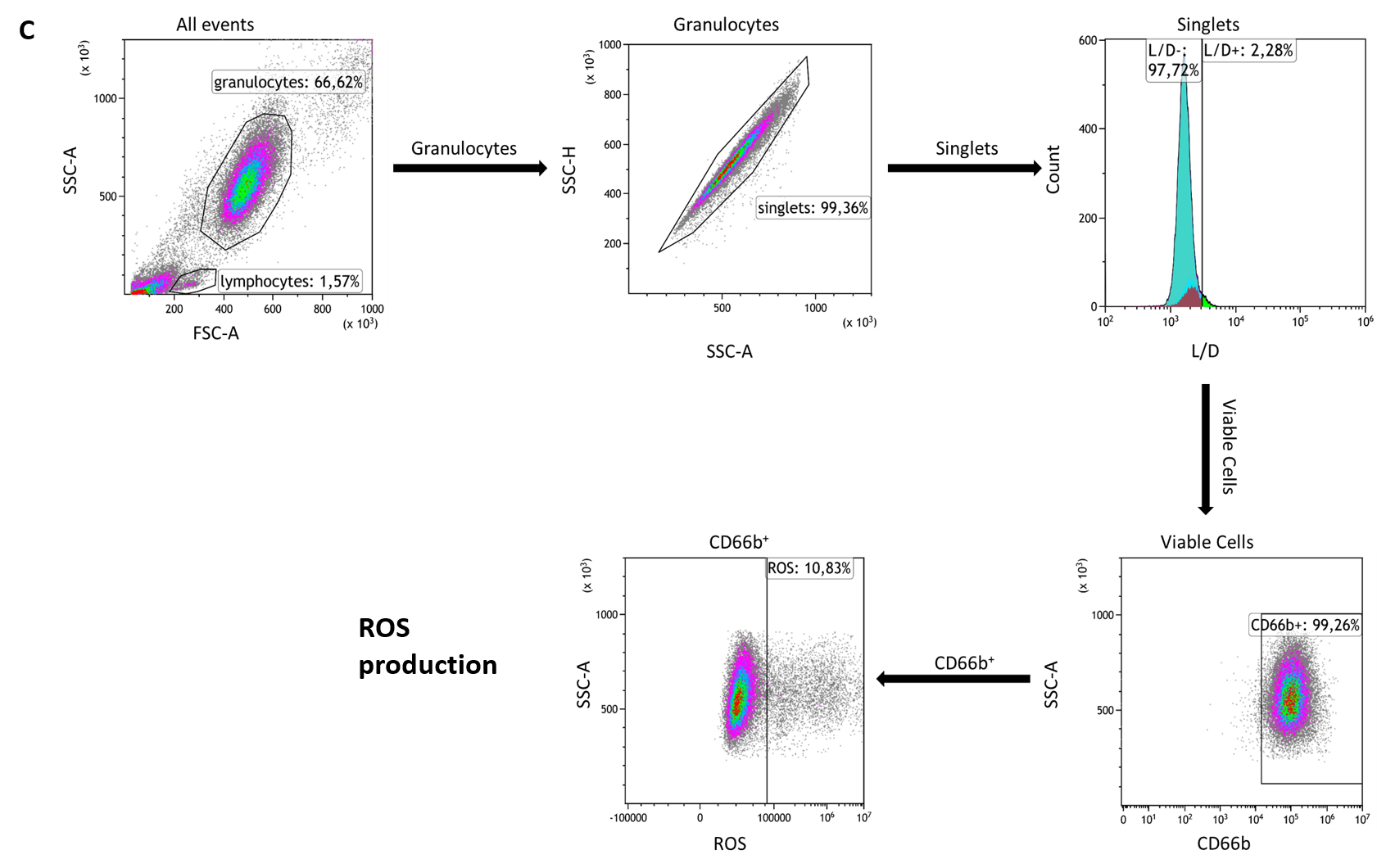

Supplement: S4 Fig — Debris was excluded from isolated granulocytes by light scatter properties and dead granulocytes were excluded by Live/Dead staining. Granulocytes were identified by CD66 expression and analyzed for ROS expression. Fluorescence positivity was determined for ROS. (TIF) [file ppat.1012315.s004.tif]

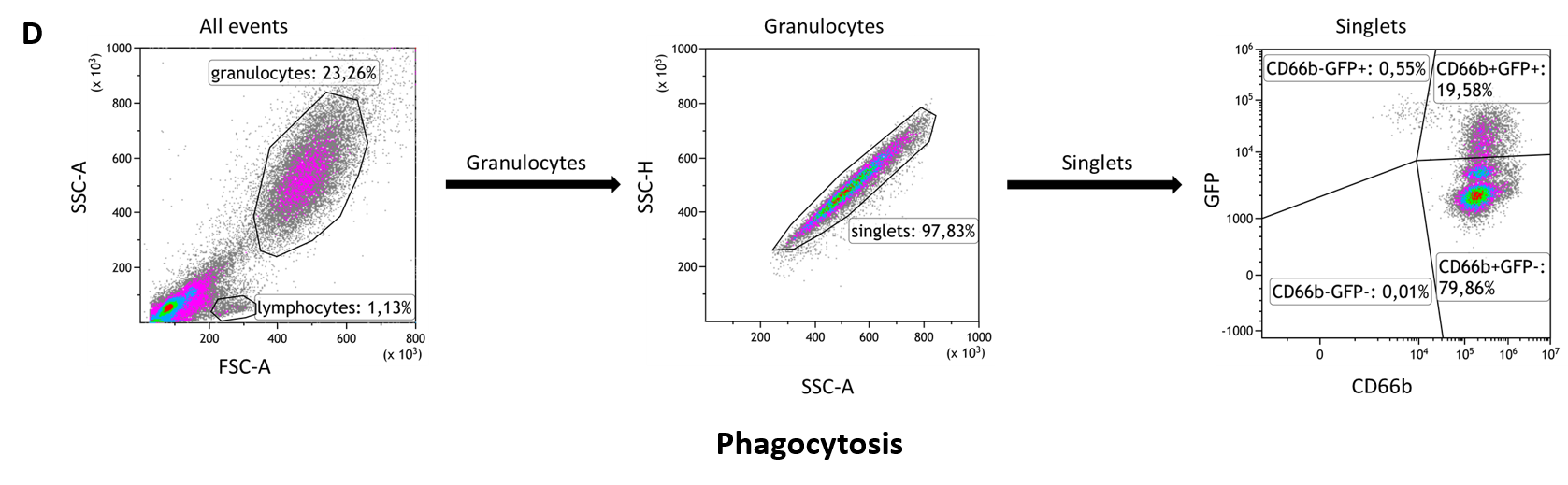

Supplement: S5 Fig — Isolated granulocytes are selected according to their forward and side scattering and single lymphocytes were gated based on SSC-A, and SSC-H properties. Conidia phagocytosed by PMNs are identified as CD66b+GFP+. (TIF) [file ppat.1012315.s005.tif]
