# Supplementary material for: CD56-mediated activation of human natural killer cells is triggered by Aspergillus fumigatus galactosaminogalactan
Source: PLoS Pathog. 2024 Jun 18;20(6):e1012315. doi: 10.1371/journal.ppat.1012315 (PMC11216564; doi:10.1371/journal.ppat.1012315)
Supplement: S1 Table — X indicates that the compound was used for the respective condition. A total volume of 200 μL was used for all conditions. Abbreviations: gt = germ tubes, PMA = Phorbol-12-myristate-13-acetate, RPMI = Roswell Park Memorial Institute medium, n/a = not applicable. (DOCX) [file ppat.1012315.s006.docx]

|  | **NK cells** | **Af293 gt** | **Δ*uge3* gt** | **Δ*agd3* gt** | **Agd3** | **Ega3** | **Sph3** | **PMA** | **Ionomycin** | **RPMI+10 % FCS** |
| --- | --- | --- | --- | --- | --- | --- | --- | --- | --- | --- |
| **Stock concentration** | 2*10^6^ cells/mL | 2*10^7^ gt | 2*10^7^ gt | 2*10^7^ gt | 132 µM | 596 µM | 568 µM | 1 mg/mL | 1 mg/mL | n/a |
| **Final concentration** | 1*10^6^ cells/mL | 4*10^6^ gt/mL | 4*10^6^ gt/mL | 4*10^6^ gt/mL | 0.1 µM | 1 µM | 2 µM | 25 ng/mL (pre-dilution 1:1000) | 1 µg/mL (pre-dilution 1:10) | n/a |
| **Unstimulated control (NK)** | 100 µL |  |  |  |  |  |  |  |  | 100 µL |
| **NK+Δ*uge3*** | 100 µL |  | 25 µL |  |  |  |  |  |  | 75 µL |
| **NK+Af293** | 100 µL | 25 µL |  |  |  |  |  |  |  | 75 µL |
| **NK+Af293+Sph3** | 100 µL | 25 µL |  |  |  |  | 25 µL |  |  | 50 µL |
| **NK+Af293+Ega3** | 100 µL | 25 µL |  |  |  | 25 µL |  |  |  | 50 µL |
| **NK+Af293+Ega3+Sph3** | 100 µL | 25 µL |  |  |  | 25 µL | 25 µL |  |  | 25 µL |
| **NK+Δ*agd3*** | 100 µL |  |  | 25 µL |  |  |  |  |  | 75 µL |
| **NK+Δ*agd3*+Agd3** | 100 µL |  |  | 25 µL | 25 µL |  |  |  |  | 50 µL |
| **NK+Δ*agd3*+Sph3** | 100 µL |  |  | 25 µL |  |  | 25 µL |  |  | 50 µL |
| **NK+Δ*agd3*+Agd3+Ega3** | 100 µL |  |  | 25 µL | 25 µL | 25 µL |  |  |  | 25 µL |
| **NK+Agd3+Ega3+Sph3 (all enzymes)** | 100 µL |  |  |  | 25 µL | 25 µL | 25 µL |  |  | 25 µL |
| **NK+PMA+Ionomycin** | 100 µL |  |  |  |  |  |  | 5 µL | 2 µL | 75 µL |

|  | **NK cells** | **Af293 gt** | **Δ*uge3* gt** | **Δ*agd3* gt** | **Agd3** | **Ega3** | **Sph3** | **PMA** | **Ionomycin** | **RPMI+10 % FCS** |
| --- | --- | --- | --- | --- | --- | --- | --- | --- | --- | --- |
| **Stock concentration** | 2*10^6^ cells/mL | 2*10^7^ gt | 2*10^7^ gt | 2*10^7^ gt | 132 µM | 596 µM | 568 µM | 1 mg/mL | 1 mg/mL | n/a |
| **Final concentration** | 1*10^6^ cells/mL | 4*10^6^ gt/mL | 4*10^6^ gt/mL | 4*10^6^ gt/mL | 0.1 µM | 1 µM | 2 µM | 25 ng/mL | 1 µg/mL | n/a |
| **Unstimulated control (NK cells)** | X |  |  |  |  |  |  |  |  | X |
| **NK+Δ*uge3*** | X |  | X |  |  |  |  |  |  | X |
| **NK+Af293** | X | X |  |  |  |  |  |  |  | X |
| **NK+Af293+Sph3** | X | X |  |  |  |  | X |  |  | X |
| **NK+Af293+Ega3** | X | X |  |  |  | X |  |  |  | X |
| **NK+Af293+Ega3+Sph3** | X | X |  |  |  | X | X |  |  | X |
| **NK+Δ*agd3*** | X |  |  | X |  |  |  |  |  | X |
| **NK+Δ*agd3*+Agd3** | X |  |  | X | X |  |  |  |  | X |
| **NK+Δ*agd3*+Sph3** | X |  |  | X |  |  | X |  |  | X |
| **NK+Δ*agd3*+Agd3+Ega3** | X |  |  | X | X | X |  |  |  | X |
| **NK+Agd3+Ega3+Sph3 (all enzymes)** | X |  |  |  | X | X | X |  |  | X |
| **NK+PMA+Ionomycin** | X |  |  |  |  |  |  | X | X | X |
