# Supplementary material for: CD56-mediated activation of human natural killer cells is triggered by Aspergillus fumigatus galactosaminogalactan
Source: PLoS Pathog. 2024 Jun 18;20(6):e1012315. doi: 10.1371/journal.ppat.1012315 (PMC11216564; doi:10.1371/journal.ppat.1012315)
Supplement: S2 Table — (DOCX) [file ppat.1012315.s007.docx]

| Antibodies | Source | Identifier |
| --- | --- | --- |
| Anti-human CD3 PE (mouse IgG1) | BD Pharmingen, San Jose, California USA | Cat#555333, Clone: monoclonal UCHT1; RRIDAB_395740 |
| Anti-human CD3 PE-Vio615 (recombinant human IgG1, REAfinity) | Miltenyi Biotec, Bergisch Gladbach, Germany | Cat#130-114-520, Clone: monoclonal  REA613;  RRIDAB_2726688 |
| Anti-human CD3 APC-Vio770 (recombinant human IgG1, REAfinity) | Miltenyi Biotec, Bergisch Gladbach, Germany | Cat# 130-113-136, Clone: monoclonal  REA613; RRIDAB_2725964 |
| Anti-human CD14 APC-Vio770 (mouse IgG2aκ) | Miltenyi Biotec, Bergisch Gladbach, Germany | Cat# 130-113-144, Clone: monoclonal  TÜK4; RRIDAB_2725972 |
| Anti-human CD56 FITC (mouse IgG1) | BD Pharmingen, San Jose, California USA | Cat#562794, Clone: monoclonal B159; RRIDAB_2737799 |
| Anti-human CD56 FITC (recombinant human IgG1, REAfinity) | Miltenyi Biotec, Bergisch Gladbach, Germany | Cat# 130-114-549, Clone: monoclonal  REA196; RRIDAB_2726695 |
| Anti-CD56 APC (mouse IgG) | CliniSciences, Nanterre, France | NB-22-63149-20  Clone: MEM188 |
| Anti-human CD66b PE-Vio770 (recombinant human IgG1, REAfinity) | Miltenyi Biotec, Bergisch Gladbach, Germany | Cat# 130-119-768, Clone: monoclonal  REA306; RRIDAB_2751829 |
| Anti-human CD69 PE-Vio615 (recombinant human IgG1, REAfinity) | Miltenyi Biotec, Bergisch Gladbach, Germany | Cat# 130-112-617, Clone: monoclonal  REA824; RRIDAB_2659073 |
| Anti-human CD69 PE-Vio770 (recombinant human IgG1, REAfinity) | Miltenyi Biotec, Bergisch Gladbach, Germany | Cat# 130-112-615, Clone: monoclonal  REA824; RRIDAB_2659075 |
| Anti-human CD69 PerCP (mouse IgG1) | Biolegend, San Diego, CA, USA | Cat#310928, Clone: monoclonal FN50; RRIDAB_10679124 |
| Anti-human CD107a PE (recombinant human IgG1, REAfinity) | Miltenyi Biotec, Bergisch Gladbach, Germany | Cat# 130-111-621, Clone: monoclonal  REA792; RRIDAB_2654474 |
| Anti-human IFN-γ PE (recombinant human IgG1, REAfinity) | Miltenyi Biotec, Bergisch Gladbach, Germany | Cat# 130-113-498, Clone: monoclonal  REA600; RRIDAB_ 2733717 |
